# Biologization of Collagen-Based Biomaterials Using Liquid-Platelet-Rich Fibrin: New Insights into Clinically Applicable Tissue Engineering

**DOI:** 10.3390/ma12233993

**Published:** 2019-12-02

**Authors:** Sarah Al-Maawi, Carlos Herrera-Vizcaíno, Anna Orlowska, Ines Willershausen, Robert Sader, Richard J Miron, Joseph Choukroun, Shahram Ghanaati

**Affiliations:** 1 Department for Oral, Cranio-Maxillofacial, and Facial Plastic Surgery, Frankfurt Orofacial Regenerative Medicine (FORM) Lab, University Hospital Frankfurt Goethe University, 60590 Frankfurt am Main, Germany; sarah.al-maawi@kgu.de (S.A.-M.); maxilofacialchv@gmail.com (C.H.-V.); a.b.orlowska@gmail.com (A.O.); ineswill@me.com (I.W.); R.Sader@em.uni-frankfurt.de (R.S.); joseph@a-prf.com (J.C.); 2 Department of Orthodontics and Orofacial Orthopedics, University of Erlangen, 91052 Erlangen, Germany; 3 Department of Periodontology, University of Bern, 3004 Bern, Switzerland; richard.miron@zmk.unibe.ch; 4 Private practice, Pain Therapy Center, 06100 Nice, France

**Keywords:** collagen, leukocytes, platelets, platelet-rich fibrin, tissue engineering, centrifugation, liquid-PRF, LSCC

## Abstract

Platelet-rich fibrin (PRF) is a blood concentrate derived from venous blood that is processed without anticoagulants by a one-step centrifugation process. This three-dimensional scaffold contains inflammatory cells and plasma proteins entrapped in a fibrin matrix. Liquid-PRF was developed based on the previously described low-speed centrifuge concept (LSCC), which allowed the introduction of a liquid-PRF formulation of fibrinogen and thrombin prior to its conversion to fibrin. Liquid-PRF was introduced to meet the clinical demand for combination with biomaterials in a clinically applicable and easy-to-use way. The aim of the present study was to evaluate, ex vivo, the interaction of the liquid-PRF constituents with five different collagen biomaterials by histological analyses. The results first demonstrated that large variability existed between the biomaterials investigated. Liquid-PRF was able to completely invade Mucograft^®^ (MG; Geistlich Biomaterials, Wolhusen, Switzerland) and to partly invade Bio-Gide^®^ (BG; Geistlich Biomaterials, Wolhusen, Switzerland) and Mucoderm^®^ (MD; Botiss Biomaterials, Berlin, Germany), and Collprotect^®^ (CP; Botiss Biomaterials, Berlin, Germany) showed only a superficial interaction. The BEGO^®^ collagen membrane (BCM; BEGO Implant Systems) appeared to be completely free of liquid-PRF. These results were confirmed by the different cellular penetration and liquid-PRF absorption coefficient (PAC) values of the evaluated membranes. The present study demonstrates a system for loading biomaterials with a complex autologous cell system (liquid-PRF) in a relatively short period of time and in a clinically relevant manner. The combination of biomaterials with liquid-PRF may be clinically utilized to enhance the bioactivity of collagen-based biomaterials and may act as a biomaterial-based growth factor delivery system.

## 1. Introduction

The function of biomaterials in regenerative medicine is to support soft and hard tissue regeneration through material-induced tissue reactions. When looking for the “ideal” biomaterial, several requirements must be considered, including tissue compatibility, physiochemical stability, the rates of vascularization and degradation, and the biomaterial-specific immune response [1]. Recently, various collagen-based biomaterials were introduced for use in guided bone and tissue regeneration (GBR/GTR). These materials are mostly resorbable, naturally derived collagenous biomaterials mainly with xenogeneic origins. Previous in vivo experiments evaluated the cellular reaction towards collagen-based biomaterials after subcutaneous implantation [2]. Different types of tissue reactions were observed, including the induction of mononuclear cells (MNCs), such as monocytes, lymphocytes, macrophages, and fibroblasts, or signs of a foreign body reaction, which is characterized by the formation of additional multinucleated giant cells (MNGCs) [2,3].

In vivo evaluation of a porcine-derived bilayer collagen membrane showed stepwise integration, inducing only MNCs, over a 60-day period [4]. Analysis of the vascularization rate revealed a mild vascularization pattern without material penetration, which emphasizes the ability of collagen-based biomaterials to integrate within the implantation region without undergoing transmembranous vascularization [4,5]. Additional investigations evaluating porcine-derived collagenous membranes of different thicknesses and physiochemical compositions revealed the presence of MNGCs. In these cases, the occurrence of MNGCs was associated with an enhanced vascularization rate and disintegration of the membranes accompanied by premature ingrowth of the peri-implantation region into the central region of the biomaterials [6,7]. These observations showed that although all biomaterials were derived from the same origin, different cellular reactions were induced based on their processing techniques and physicochemical properties.

The role of collagen-based biomaterials in GBR has been explored in vivo, showing the crucial influence of new bone formation when comparing bony defects covered with a collagen membrane to those without collagen membranes [8]. However, the biomaterial characteristics may influence the underlying bone area. Several investigations have confirmed the function of different collagen-based biomaterials as bioactive components involved in bone regeneration in combination with different bone substitutes [9]. These findings are relevant for clinical applications of pure biomaterials [10,11]. Moreover, previously published, in vivo cell-based, tissue engineering studies that considered the use of biological concepts, such as the combination of biomaterials with primary human cells or pre-seeding with monocytes, have demonstrated their effects on the vascularization patterns in the biomaterials at hand, and the consequently enhanced regenerative capacities [12]. However, cell-based tissue engineering is a sensitive technique that requires special conditions, which are mostly not available in clinical settings. A clinically applicable system was, therefore, highly needed to reap the benefits of these observations.

This blood concentrate is derived from the patient’s peripheral blood after a one-step centrifugation without anticoagulants to generate a platelet and leucocyte-rich matrix. The presence of platelets, leucocytes, and fibrin was previously shown to be essential for wound healing [13,14]. In addition to the potential of leucocytes to influence angiogenesis and lymphomagenesis, this fibrin network, containing leucocytes and platelets, is a source of cytokines and growth factors, which are the main players in the process of wound healing [15]. The use of specific plastic tubes favors nonclotting platelet-rich fibrin (PRF) and results in the development of a liquid-PRF-based matrix (liquid-PRF) produced without the need for anticoagulants. Previously, a systematic study investigated the influence of the applied relative centrifugal force (RCF) on the composition and bioactivity of PRF matrices [16]. In that study, it was shown that liquid-PRF generated with the lowest RCF contained the highest concentration of platelets and leukocytes and released significantly higher concentrations of different growth factors compared to two other liquid-PRF matrices prepared at higher RCF [16]. Therefore, the low-speed centrifugation concept (LSCC) was described to standardize the centrifugation of blood concentrates.

The combination of autologous biological products; i.e., PRF-based matrices with xenogeneic biomaterials, has been of great clinical interest. The application of PRF matrices with different centrifugation protocols in combination with collagen-based biomaterials was previously described in several clinical studies, especially in periodontology [17]. However, the results are not consistent. Some studies showed a positive effect of the addition of PRF to collagen-based biomaterials, while others did not show any effect [18]. It may be that the biomaterial-specific physicochemical characteristics, such as surface structure, absorption capacity, porosity, and thickness, may influence the interaction between the collagen-based biomaterial and PRF. However, to date, no published studies have evaluated the feasibility of combining collagen-based biomaterials and PRF in detail. Therefore, the aim of the present study was to evaluate the interaction of liquid-PRF with five different collagenous biomaterials by histological analyses to identify the most promising combination for clinical use. To the best of our knowledge, this was the first study to analyze the combination of different commercially available collagen-based biomaterials and liquid-PRF ex vivo. Particular attention was focused on the absorption capacities and penetration patterns of the leucocytes and platelets within the collagen-based biomaterials after liquid-PRF application, and on the interaction between collagen and fibrin.

## 2. Materials and Methods

In the present study, five different commercially available collagen-based biomaterials were used.

### 2.1. Collagen Membranes

Mucograft^®^ (MG; Geistlich Biomaterials, Wolhusen, Switzerland) is a bilayered collagen matrix derived from porcine peritoneum and skin. The collagen is derived from a certified porcine source and is processed to purify it from genetically active constituents. The matrix consists of type I and type III collagen and is processed using standardized methods and sterilized by gamma irradiation without chemical treatment or additional cross-linking. The bilayered structure of the matrix consists of a thin, smooth, compact layer with low porosity and a three-dimensional spongy layer with higher porosity. These layers are connected using a biophysical interweaving process. The pore system of the matrix is manufactured via defined parameters and lyophilization to include both small and large pores (5–200 µm). This variously-sized pores serve as a matrix for tissue and cell adherence and stabilize the blood clot after application.

Bio-Gide^®^ (BG; Geistlich Biomaterials, Wolhusen, Switzerland) is a porcine-derived collagen membrane containing type I and III collagen without cross-linking. This bilayered membrane is manufactured using a standardized process to eliminate immunologically active constituents, microorganisms, and other residues. Collagen is obtained from a veterinary-certified porcine source and is sterilized by gamma irradiation. The pure bilayered collagen membrane consists of a smooth, thin, compact layer with low porosity and a three-dimensional, more porous spongy layer. This structure should support bone formation and inhibit connective tissue ingrowth in bony defects.

Mucoderm^®^ (MD; Botiss Biomaterials, Berlin, Germany) is a porcine dermis-based collagen matrix. The membrane is purified by a multistep cleaning method, lyophilized, and sterilized by gamma irradiation to produce a three-dimensional non-cross-linked membrane of collagen and elastin.

Collprotect^®^ (CP; Botiss Biomaterials, Berlin, Germany) is a bilayered cross-linked collagen membrane containing type I and type III collagen and elastin. The membrane is obtained from porcine dermis. Purification methods, such as multistep cleaning, lyophilization, and gamma irradiation, were performed to generate a stable three-dimensional bilayered collagen membrane.

BEGO^®^ collagen membrane (BCM; BEGO Implant Systems) is a non-cross-linked, stratified membrane obtained from porcine pericardium. Donor animals are selected in a veterinary-controlled porcine source. The extracted collagen is treated by a controlled purification process, lyophilized, and sterilized with ethylene oxide gas.

### 2.2. Scanning Electron Microscopy

The ultrastructure of the collagen-based biomaterials was examined under a DSM 962 SEM (scanning electron microscope, Zeiss, Oberkochen, Germany) operated with a LaB6 cathode, which can be used to obtain medium resolution photographs. After sputter coating the samples with 20–30 nm gold in a cold sputter unit, images were taken at 10 keV acceleration energy and an object distance of approximately 12.0 cm. The SEM images were analyzed with the Kontron KS 300 image analysis program (Carl Zeiss Vision, Eching, Germany).

### 2.3. Liquid-PRF Preparation

The application of PRF in this study was approved by the responsible Ethics Commission of the state of Hessen, Germany (265/17). Liquid-PRF was obtained from venous blood without additional anticoagulants as previously described [16]. Three healthy volunteers, aged 18–50 years with no anticoagulant usage, donated peripheral blood for this study after informed consent was obtained. Four sterile, 10 mL plastic tubes (Process for PRF, France, radius 110 mm) full of blood were obtained from each participant. The filled tubes were immediately placed in the centrifuge, with two tubes placed directly opposite each other for balance during centrifugation. The liquid centrifugation protocol (10 mL, 600 rpm, 44 × *g* for 8 min) according to LSCC was performed in a programmed centrifuge (Duo centrifuge; PROCESS for PRF, Nice, France). The tubes that were used did not contain any type of anticoagulants in order to avoid interference with platelet activity. After centrifugation, the tubes showed a multiphasic liquid that contained an upper phase of a yellow–orange-colored liquid (liquid-PRF) and a lower red phase of the remaining blood constituents. The tubes were carefully opened to avoid mixing the phases, and 1–2 mL of the upper liquid (liquid-PRF) was collected using a 10 mL syringe with a needle (B Braun inject^®^) and transferred to a flask.

### 2.4. Liquid-PRF Application

Four samples (10 × 10 mm) were prepared from each collagenous biomaterial using scissors. The material sample was placed on a 4 × 6 cell culture plate, and the prepared 500 µL liquid-PRF was applied to the collagen-based biomaterial samples, covering the entire sample. The membranes were kept in liquid-PRF until clotting (15 min at room temperature). Then, the samples were fixed in 4% formaldehyde for 24 h.

### 2.5. Histological Preparation

The fixed samples were processed according to previously described techniques [5]. Briefly, after processing, six 3–4 µm sections were cut from each sample using a rotary microtome (Leica RM2255; Wetzlar, Germany). Next, the samples were stained with different histochemical stains. The first slice was stained with hematoxylin and eosin (H&E) according to standard protocols, and the other five slices were stained with Azan–Mallory, Masson–Goldner, and Giemsa.

### 2.6. Histological Analysis

Morphological and qualitative histological analyses of the prepared slices were performed using a Nikon ECLIPSE 80i microscope (Tokyo, Japan). We focused on the interaction with liquid-PRF, particularly the locations and distributions of leucocytes and platelets, and the liquid-PRF invasion of the biomaterials. Additionally, histological photographs were captured using a Nikon DS-Fi1/Digital camera with a digital sight control unit.

### 2.7. Histomorphometrical and Statistical Analysis

Four Giemsa-stained slides of each biomaterial were used for the histomorphometric analysis of cellular penetration. Total scans were created using a light microscope (Nikon, Tokyo, Japan) with a scanning table (Prior, Rockland, MA, USA) connected to a DS-Fi/1 digital camera (Nikon) and a PC running NIS Elements AR software (version 4.1; Nikon). A total scan is a merge of 50–100 single images of the region of interest. Subsequently, the thickness of the biomaterials was measured (in µm) using the measurement tools in NIS Elements. The penetration distance into the biomaterials was measured in µm. Cellular penetration into the central region (thickness/2) of the biomaterial was set to 100%. 

### 2.8. Fluid Absorption Capacity

The capacity of each biomaterial to absorb liquid-PRF was measured using a gravimetric method and named the PRF absorption coefficient (PAC). This method was based on the difference in sample weight before versus after the application of a specific liquid. In this experiment, two different liquids were tested: distilled water and liquid-PRF. Three segments of 0.8 mm diameter (*d*) from each biomaterial were extracted using biopsy punches (SmithKline Beecham, NW, UK) weighed in a dry state (W0) and then placed in 24-well cell culture plates. Five hundred microliters of liquid-PRF were deposited on top of the biomaterials; incubation for 15 min at room temperature was then performed—until clotting. The weight of the biomaterials after incubation was registered (W1), and the absorption coefficient was calculated using the following formula: PAC = (W1 − W0)/W0. The same procedure was repeated with distilled water as a control measurement (WAC).

### 2.9. Statistical Analysis

The results are expressed as the means and standard deviations. GraphPad Prism 7 (GraphPad Software, Inc., La Jolla, USA) was used to generate charts and perform statistical analyses using one-way analysis of variance with Tukey’s multiple comparisons test (α = 0.05). Values were considered significant if *p* < 0.05 (*) and highly significant at *p* < 0.01 (**), *p* < 0.001 (***), and *p* < 0.0001 (****).

## 3. Results

The results revealed the structural details of the biomaterials before combination with PRF to evaluate differences in the physical structures ex vivo. This may be relevant to understanding the interaction between the material and liquid-PRF, as described in the following sections.

### 3.1. Ultrastructure of the Collagen-Based Biomaterials without PRF

**Figure 1:**
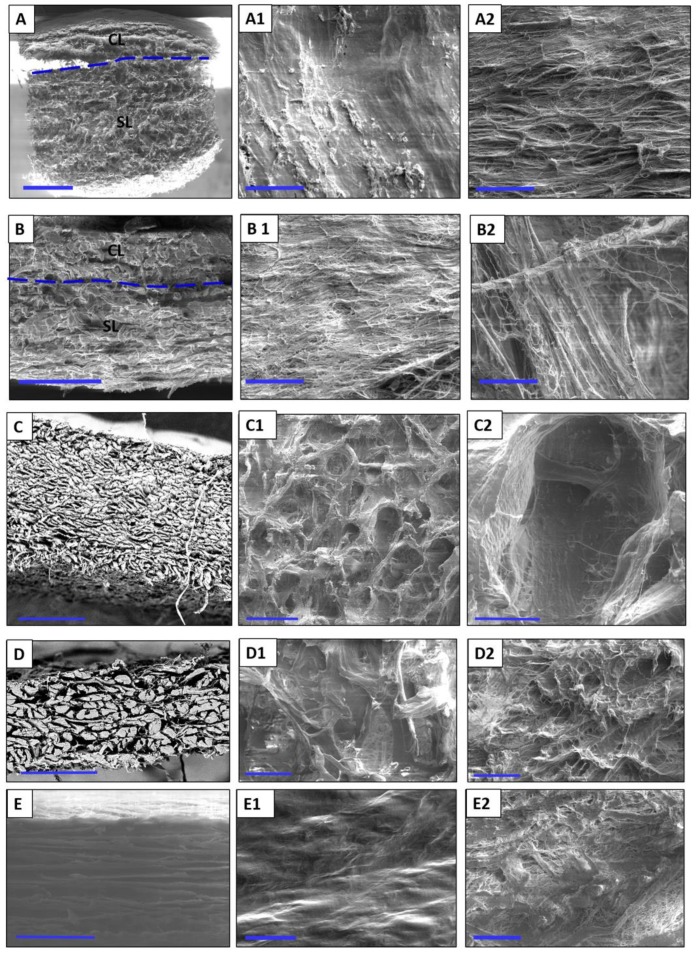
Representative scanning electron microscopy images showing the ultrastructure of the evaluated biomaterials. (**A**) MG in a cross section (×25, 10 Kv, scale bar = 1 mm); (**A1**) the smooth surface of MG (×500, 5 kV, scale bar = 50 µm); (**A2**) the rough surface of MG (×500, 5 kV, scale bar = 50 µm). (**B**) Cross section of BG (×200, 10 kV, scale bar = 200 µm); (**B1**) the smooth surface of BG (×500, 5 kV, scale bar = 50 µm); (**B2**) the rough surface of BG (×500, 5 kV, scale bar = 50 µm). (**C**) Cross section of MD (×50, 15 kV, scale bar = 500 µm); (**C1**) the surface of MD (×100, 5 Kv, scale bar = 200 µm); (**C2**) the surface of MD at a higher magnification (×500, 5 kV, scale bar = 50 µm). (**D**) Cross section of CP (×200, 15 kV, scale bar = 200 µm); (**D1**) the rough side of CP (×500, 5 kV, scale bar = 50 µm); (**D2**) the smooth side of CP (×500, 5 kV, scale bar = 50 µm); (**E**) cross section of BCM (×500, 15 kV, scale bar = 50 µm); (**E1**) smooth surface of BCM (×500, 5 kV, scale bar = 50 µm); (**E2**) the rough surface of BCM (×500, 5 kV, scale bar = 50 µm).

Mucograft^®^ (MG): The structure of MG was observed in the cross section as a bilayered collagen matrix. The layers could be differentiated because of their variation in porosity. The compact layer (CL) was thinner and exhibited lower porosity than the spongy layer (SL; Figure 1A). The surface of the CL was smooth and even (Figure 1A1). In contrast, the surface of the spongy layer was more heterogeneous. Collagen fibers of different diameters were observed in a defined arrangement, which made the surface of the SL rough (Figure 1A2).

Bio-Gide^®^ (BG): In the cross section, the ultrastructure of BG showed two different layers; i.e., the CL and SL. The arrangement of the collagen fibers allowed the identification and differentiation of the layers. Most of the collagen fibers in the CL were arranged vertically, which imparted low porosity, whereas the collagen fibers within the SL were arranged in parallel horizontally direction, which contributed to its more porous appearance (Figure 1B). The surface of the CL consisted of collagen fibers that were close to each other, imparting a smooth, even surface with small intercollagenous spaces and low porosity (Figure 1B1). The SL showed a dynamic distribution of collagen fibers, which provided a more porous structure and a rather surface (Figure 1B2).

Mucoderm^®^ (MD): In the cross section, MD showed uniformly arranged collagen fibers that appeared to be tightly interwoven (Figure 1C). The surface structure exhibited a high roughness (Figure 1C1,C2). The porous system was compact and consistent.

Collprotect^®^ (CP): In cross section, the membrane appeared homogenous, with irregularly arranged collagen fibers. Different-sized pores were unequally distributed throughout the cross section (Figure 1D). The surfaces of both sides appeared to be different. The rough side exhibited a very uneven surface with upward-directed fibers (Figure 1D1). In contrast, the smooth side was comparably smooth, with fine collagen fibers that formed a slightly wavy surface (Figure 1D2).

BEGO collagen membrane (BCM): A cross section of the BCM showed parallelly-arranged collagen fibers with uniform porosity (Figure 1E). The surfaces of the membrane differed according to their morphology. The smooth surface was evenly wavy without obvious pores (Figure 1E1), whereas the rough surface was compact but more porous, with collagen fibers arranged in different directions (Figure 1E2).

### 3.2. Histological Evaluation of Material–PRF Interaction

Qualitative histological analysis of each tissues was performed, focusing on the interaction between the liquid-PR and each collagen-based biomaterial. A particular emphasis was placed on the distribution of leucocytes and platelets within the biomaterials, and the fibrin saturation.

**Figure 2:**
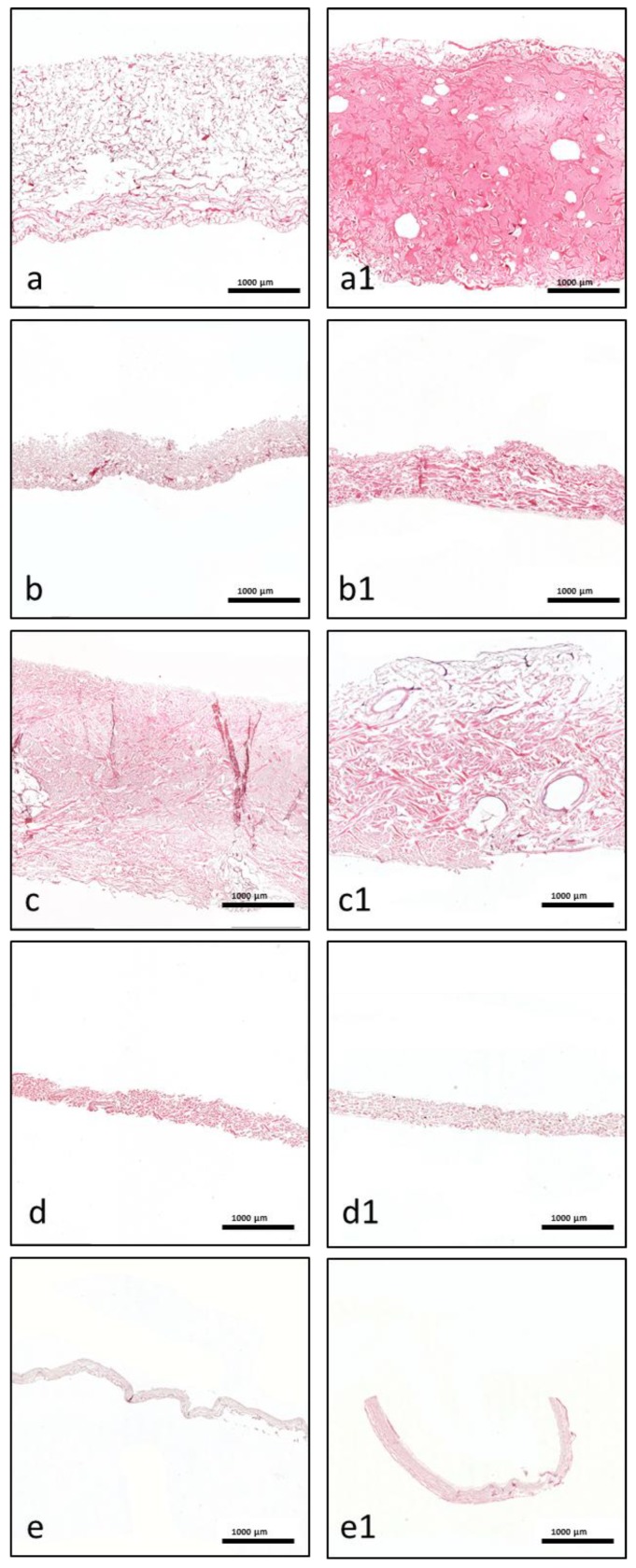
Representative histological micrographs. (**a**–**e**) Cross sections of blank membranes without liquid-PRF as a control (all pictures are total scan sections in H.E. staining, ×40 magnification, scale bar = 1000 µm). (**a1**–**e1**) Cross sections of membranes after liquid-PRF incubation (all pictures are total scan sections in hematoxylin and eosin staining, ×40 magnification, scale bar = 1000 µm). (**a**,**a1**) MG, (**b**,**b1**) BG, (**c**,**c1**) MD, (**d**,**d1**) CP, and (**e**,**e1**) BCM.

**Figure 3:**
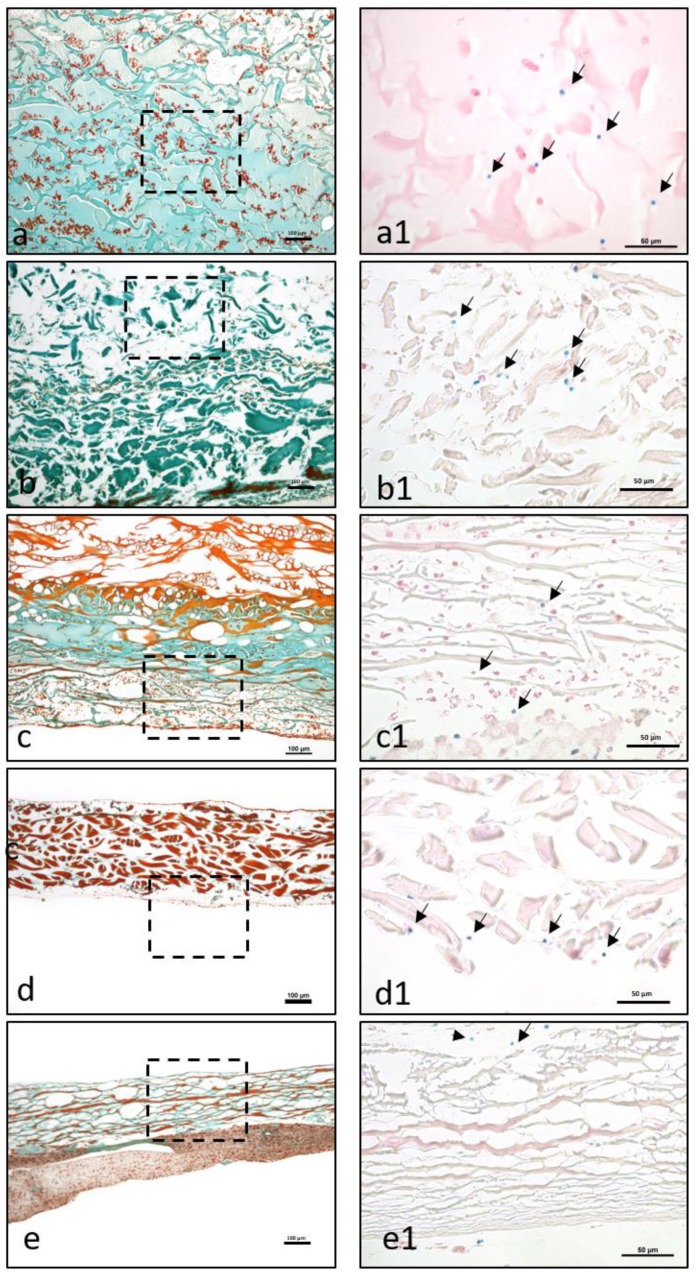
The interaction between liquid-PRF and the evaluated membranes. (**a**–**e**) The formation of PRF-clots on the membrane (**a**) or on the membrane surface (**b**–**e**). (**a**–**e**) Masson Goldner staining, ×100 magnification. Dashed lines refer to the area, which is presented at a higher magnification in (**a1**–**e1**). (**a1**–**e1**) A higher magnification of the membrane-cell interaction in the different membrane types. Arrows point to the cell localization in the different membranes. Giemsa staining at 400× magnification. (**a**,**a1**) MG, (**b**,**b1**) BG, (**c**,**c1**) MD, (**d**,**d1**) CP, and (**e**,**e1**) BCM.

Mucograft ^®^ (MG): Liquid-PRF was detectable between the collagen fibers of the matrix, and liquid-PRF had invaded the entire collagen matrix; i.e., the SL and CL (Figure 2a,a1). Additionally, the pores appeared to be mostly full of liquid-PRF, and the leucocytes and platelets had reached the central region of the collagen matrix and were evenly distributed. These cells were clearly observable in the superficial layer and within the core of the membrane. A PRF-clot was observable on the surface of the matrix (Figure 3a,a1).

Bio-Gide^®^ (BG): After liquid-PRF application, the bilayered collagen membrane showed a variable distribution pattern. Leucocytes and platelets were located between the collagen fibers on the superficial parts of the CL (Figure 2b,b1). In contrast, the SL appeared to be largely free of cells. In addition, there were no leucocytes or platelets in the central region of the collagen membrane. While a stable, continuous liquid-PRF clot was located adjacent to the surface of the membrane on the SL (Figure 3b,b1), only a sporadic liquid-PRF was observed on the surface of the CL.

Mucoderm^®^ (MD): Both sides of the biomaterial interacted with the liquid-PRF matrix. However, the interaction was only observed on the very superficial layers of the membrane (Figure 2c,c1). There was almost no liquid-PRF invasion within the superficial layers, although some single leucocytes and platelets were observed (Figure 3c,c1). These cells did not enter the material body or reach the center. Accordingly, most of the collagenous material contained no fibrin or leucocytes. In addition, some PRF clot formation was observed on the collagen membrane surface.

Collprotect^®^ (CP): Both sides of the membrane had leucocytes and platelets between the collagen fibers of the superficial layer (Figure 2d,d1). However, the central region of the membrane was free of leucocytes and platelets (Figure 3d,d1). Liquid-PRF did not invade the membrane; therefore, no fibrin was observed in any part of the membrane; however, a PRF clot did form on the surface of the membrane.

BEGO^®^ collagen membrane (BCM): The liquid-PRF did not enter the collagen membrane. No leucocytes or platelets were found in any region of the membrane (Figure 2e,e1), and no fibrin was observed within the membrane. Additionally, the membrane appeared to be embedded within a PRF clot, without allowing the liquid-PRF to enter the membrane central region (Figure 3e,e1).

### 3.3. Histomorphometrical Results of the PRF Penetration Pattern within the Different Collagen-Based Biomaterials

**Figure 4:**
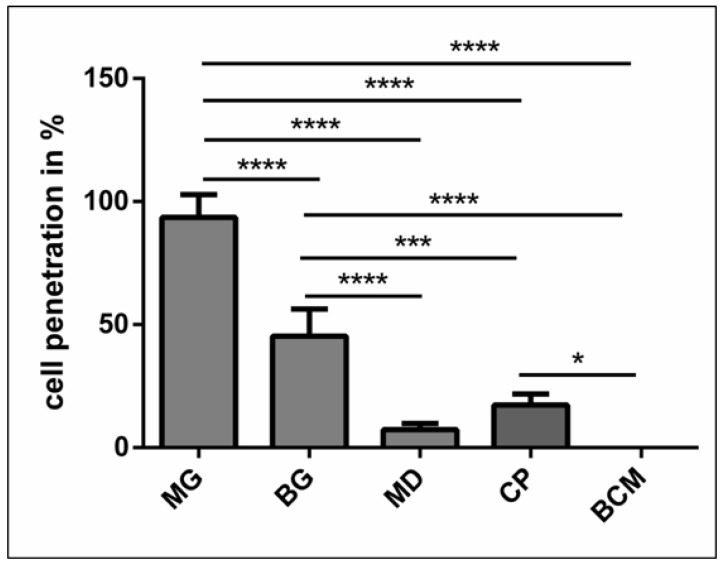
Statistical analysis of the cell penetration in percent with regard to the biomaterial thickness. Statistically significance at *p* < 0.05 (*) and high significance at *p* < 0.01 (**), *p* < 0.001 (***) and *p* < 0.0001 (****).

Cellular penetration of the platelets and leukocytes into the different biomaterials was evaluated histomorphometrically. The analysis revealed that MG showed the highest percentage of cellular penetration, and compared to all other evaluated materials, the difference was highly significant (*p* < 0.0001 ****). The cellular penetration of BG was significantly higher than that of MD (*p* < 0.0001 ****), CP (*p* < 0.001 ***), and BMC (*p* < 0.0001 ****). CP showed significantly higher cellular penetration than BMC (*p* < 0.05 *). However, there were no statistically significant differences between MD and CP or MD and BCM (Figure 4).

### 3.4. Water and Liquid-PRF Absorption Coefficient (WAC/PAC) in the Different Collagen-Based Biomaterials

Similar absorption patterns were observed for both WAC and PAC. In the case of the WAC, MG absorbed a significantly higher amount of water compared to BG (**** *p* < 0.0001), MD (**** *p* < 0.0001), CP (**** *p* < 0.0001), and BCM (**** *p* < 0.0001). No statistically significant differences were found between the other groups

**Figure 5:**
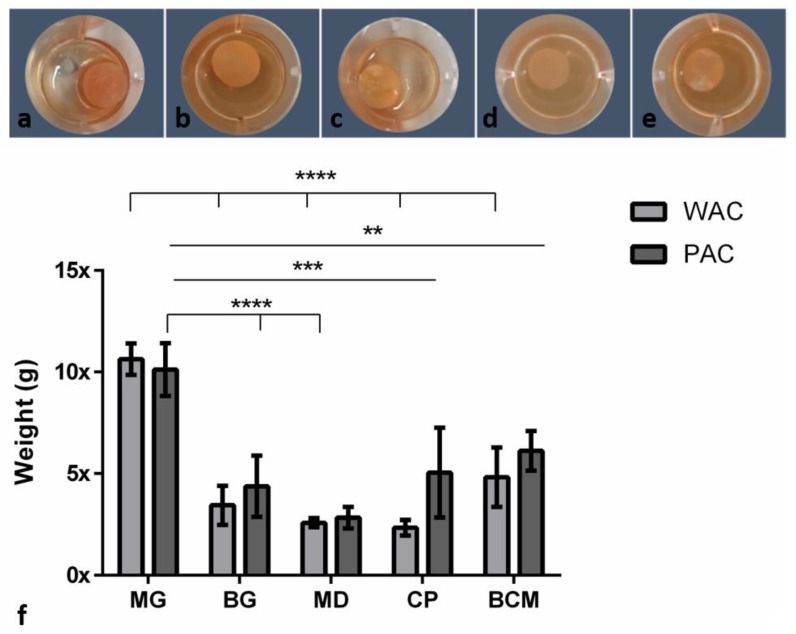
(**a**–**e**) The different membranes incubated in liquid-PRF: (**a**) MG, (**b**) BG, (**c**) MD, (**d**) CP, and (**e**) BCM. (**f**) Water absorption coefficient (WAC) and liquid-PRF absorption coefficient (PAC). Differences were considered statistically significant at *p* < 0.05 (*) and as highly significant at *p* < 0.01 (**), *p* < 0.001 (***) and *p* < 0.0001 (****).

For the PAC, the results demonstrated that MG can absorb a high content of liquid-PRF components and increase its original weight up to 10 times (10.12 ± 1.29). BG increased its weight four times (4.37 ± 1.50) after being immersed in liquid-PRF. MD showed the lowest PAC with an increase of two times its original weight (2.83 ± 0.53). CP exhibited a middle range of PAC values with an increase of five times its weight (5.05 ± 2.21) after immersion in liquid-PRF. In the case of BCM, the measurements showed an increase of six times its original weight (6.12 ± 0.97). Thereby, MG absorbed significantly more liquid-PRF compared to BG (**** *p* < 0.0001), MD (**** *p* < 0.0001), CP (*** *p* < 0.001), and BCM (** *p* < 0.01). No statistical significant differences were found between the other groups (Figure 5).

### 3.5. Classification of Collagen Liquid-PRF Interaction

**Figure 6:**
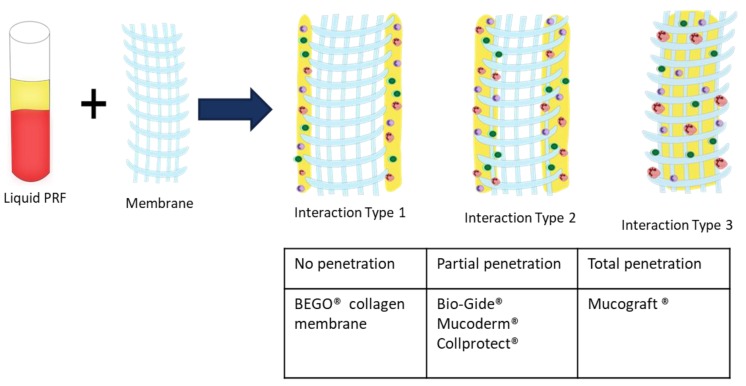
Classification of collagen-based biomaterials based on their interactions with liquid-PRF.

The histological observations allowed the classification of collagen-based biomaterials based on their interactions with liquid-PRF. Thereby, three different interaction types were observed. MG allowed total penetration of liquid-PRF into its central region and represented reaction type I (class I). MD, CP, and BG showed only some penetration of PRF into the collagen-based biomaterials. However, the central region was free of liquid-PRF. These materials represented interaction type II (class II). Finally, BCM was completely occlusive over the penetration of liquid-PRF, and therefore represented a further, type III interaction (class III) (Figure 6).

## 4. Discussion

The development of biomaterials for use in cell-based tissue engineering has focused on the in vitro preculturing of biomaterials. This technique showed enhanced vascularization after the addition of isolated cells, such as endothelial cells, mesenchymal cells, or primary human osteoblasts, in mono or cocultures [14]. The procedure of cell isolation and preculturing of biomaterials is dependent on various factors, including harvesting human tissue, cell isolation, and cultivation under aseptic conditions in laboratories. Precultivation of biomaterials can take up to two weeks [19]. In addition, cell cultures are sensitive to viral and bacterial infection and environmental changes [20]. These factors are practical limitations and drawbacks for routine clinical use. With the introduction of PRF [13,21], scientific developments have met the requirements for clinical application; thus, cell-based tissue engineering and regeneration have become clinically applicable. In contrast to isolated cells, PRF is a complex system that includes human platelets, leukocytes, and plasma proteins within a fibrin matrix [22]. Those components are key elements in wound healing and tissue regeneration. Leukocytes promote regeneration by releasing signaling molecules involved in angiogenesis, cellular cross-talk [15,23], and cell-cell communication during bone formation [24]. Platelets are essential for wound healing and tissue regeneration, as they express numerous platelet-derived growth factors (PDGFs), such as vascular endothelial growth factor (VEGF), which promotes vascularization, and transforming growth factor-beta (TGF-β), which influences the function of cells involved in new tissue formation, such as fibroblasts [23,25,26]. Thus, the interplay between these cells leads to better performance in regeneration [27]. The so-called low-speed centrifugation concept (LSCC) leads to significant enrichment of liquid-PRF-based matrices with leukocytes and platelets [16]. Furthermore, liquid-PRF has a suitable consistency and is easy to handle. The reasoning behind combining collagen membranes with liquid-PRF is to make use of the liquid-PRF constituents, such as leukocytes, platelets, and fibrin, to support guided bone and tissue regeneration (GTR/GBR).

This study presented a histological analysis of the combination of autologous liquid-PRF matrix and xenogeneic collagen-based biomaterials. However, to date, it is unknown which type of collagen-based biomaterial is most suitable for combination with liquid-PRF. Therefore, this ex vivo study was the first to analyze the interaction pattern between PRF and different collagen-based biomaterials with the aim of understanding the capacity and suitability of biomaterials to incorporate PRF. The results are potentially useful for translation into specific clinical indications with improved regeneration potential. The results of the present study showed differences in the structural composition of the collagen membranes observed by scanning electron microscopy and variations in the interactions between the collagen-based biomaterials and liquid-PRF, supporting previous findings that the cellular-biomaterial interaction is partially determined by the structural characteristics of the biomaterial [28,29].

MG is a collagen matrix composed of a smooth and rough surface. When mixed with liquid-PRF, the collagen matrix appeared completely loaded with liquid-PRF, including leukocytes, platelets and fibrin matrix, which were evenly distributed throughout the collagen matrix. Additionally, MG showed the higher PAC of all the membranes included in this study, ratifying the histological findings. The present ex vivo results also underlined the ability of MG to serve as an “enriched” scaffold with liquid-PRF, including cells and fibrin matrix. According to the literature, one of the most common clinical indications of MG in dentistry is the coverage of periodontal recession defects by GTR. In a previous clinical study by our group, MG was used to cover skin defects to induce soft tissue regeneration in patients undergoing skin cancer removal [30]. The histological results revealed an integrated membrane, inducing a mononuclear cellular reaction and a desirable clinical appearance [30]. The interaction between the collagen-based matrix and liquid-PRF, i.e., complete enrichment of the collagen matrix with liquid-PRF, may lead to a better tissue regeneration process in clinical applications as an effect of the regeneration potential of liquid-PRF. However, further clinical studies are required.

BG is a collagen bilayered membrane with one compact and one spongy layer. In this scenario, in the cell–biomaterial interaction, liquid-PRF showed a different distribution within BG. The CL allowed cells (leukocytes and platelets) and the fibrin matrix to partially enter the membrane, whereas the SL was free of liquid-PRF. Therefore, the central region of the material showed no liquid-PRF cellular penetration. Interestingly, the SL, which was free of liquid-PRF, had a well-adhered liquid-PRF clot on the surface. The results confirm the primary clinical use for BG, which is GBR. As stated by the manufacturer, the SL should face the bony defect, and the CL should be adapted to the soft tissue. Clinically, by applying the “enriched” membrane to a bony defect, BG would preserve its clinical indication, and the liquid-PRF clot on the surface would face the bony defect to support its regeneration.

In comparison, MD and CP allowed less liquid-PRF intake, as observed histologically and by the PAC measurements. These collagen-based biomaterials had leukocytes and platelets only in the most superficial layer. Those cellular–biomaterial interactions could be explained by the biomaterials’ compact structures with fibers of larger size, which were presumably impeding the expansion of biomaterials and reducing their PAC properties.

MD contained liquid-PRF fibrin between the most superficial collagen fibers, and both biomaterials showed a stable liquid-PRF clot on their surface. These materials have been used clinically in GBR/GTR. In this scenario, the result once again confirmed the membranes’ clinical indication for GBR/GTR due to the observed liquid-PRF formation of a PRF clot on their surface. Remarkably, in a previous in vivo study by our group using a subcutaneous model in Wistar rats, a pathological cellular reaction lead by MNGCs was observed on the surface of these liquid-PRF collagen-based biomaterials. The implications of an enriched collagen biomaterial, i.e., covered with a liquid-PRF clot, as to whether the inflammatory cellular reaction would be modulated, is a topic of research that requires further investigation.

The final biomaterial tested, BCM, appeared to be surrounded by a large liquid-PRF clot, and histologically no leukocytes, platelets, or fibrin were observed invading the membrane. Nevertheless, the PAC measurements of BMC showed an absorbing coefficient of five. These results suggest that BMC and the aforementioned collagen membranes may have also absorbed other components of liquid-PRF, which were not the focus of this histological evaluation. According to the manufacturer, BCM is to be used for GBR/GTR, which is in accordance with the formation of a liquid-PRF clot on the surface of the membrane. In this context, the adherent liquid-PRF clot would interact with the surrounding tissue. The rationale behind this cellular-biomaterial interaction liquid-PRF could be related to the physicochemical characteristics of the biomaterial due to the methods used to sterilize the membranes. BCM is the only biomaterial investigated in this study that is sterilized with ethylene oxide gas [7]; the other evaluated membranes are sterilized with gamma irradiation [4,5,6], which might influence the physiochemical composition of the biomaterial and its interaction with liquid-PRF.

Remarkably, MD, CP, and BCM showed commonalities ex vivo. These collagenous materials prevented the liquid-PRF components from entering the membrane central region, leading to the formation of a PRF clot on the surfaces. Additionally, the ultrastructural analyses of the various collagen-based biomaterials showed different characteristics.

It seems that the physiochemical composition and surface properties of these biomaterials are critical elements for cellular interaction. These characteristics are probably related to the techniques used to process the biomaterials during manufacturing and the harvesting compartment. The various interaction patterns observed in this study are of great clinical relevance. Based on the results presented here, including three different interaction types (class I–III), this approach may be useful as a tool to further classify and evaluate the capacity of different collagen-based biomaterials to assess their regenerative capacities. However, this study did not provide any results about the bioactivity of the different membranes, after combination with PRF. Therefore, further studies are need to further verify this system.

Interestingly, although all of the biomaterials evaluated here are of porcine origin, variable interactions with liquid-PRF were observed. Evidently, these differences depended on the biomaterial properties, since the protocol to obtain liquid-PRF was consistent. Accordingly, the physiochemical compositions of the particular biomaterials might influence their interactions with inflammatory cells. Therefore, liquid-PRF might be a useful tool for ex vivo examination of the initial response towards novel biomaterials to predict the in vivo cellular response.

Previous in vitro studies have shown that PRF-based matrices can be considered delivery systems because of their ability to release different growth factors [16]. When combining xenogeneic biomaterials with autologous liquid-PRF, the question raised is to what extent the addition of liquid-PRF, as a dose of physiological inflammatory cells, can influence the biomaterial-related cellular reaction. It might be that the total cellular penetration of the biomaterial, including the fibrin matrix within the collagen matrix, could act as a biomaterial-based delivery system, leading to enhanced vascularization and tissue regeneration, as the regenerative potential of collagen-based biomaterials and fibrin-based liquid-PRF are used simultaneously.

Furthermore, the present study utilized a protocol of 600 RPM for 8 min to produce liquid-PRF, which was previously introduced by the low-speed centrifugation concept [16]. Additionally, a systematic study investigated the influence of reducing the RCF as a proof of LSCC by using a 3 min centrifugation time and showed similar results [31]. More recently, it was further shown that the 8 min protocol utilized in our study represents a superior and more effective means to concentrate platelets compared to many commercially available protocols [32]. Previous studies have described the role of collagen-based biomaterials as a bioactive compartment in GTR by demonstrating the effect of placing a collagen membrane on newly formed bone [8,9]. Accordingly, the combination of liquid-PRF with collagen-based biomaterials introduced in this study aimed to optimize the bioactivity of biomaterials to achieve better regeneration using a clinically applicable system. The present study proved that it is possible to load xenogeneic collagen-based biomaterials with a complex mixture of autologous cells (liquid-PRF) using a clinically applicable method in less than 15 min. Additionally, we showed a histological analysis of each distribution pattern of liquid-PRF after its application on various biomaterials. However, the present observations do not provide any information as to what extent this biologically complex system, i.e., liquid-PRF with various collagen-based biomaterials, will influence the functionality of the included cells and growth factors. Additionally, the influence of PRF on the mechanical properties of the biomaterials and the activation of the cells in PRF after contact with the biomaterial surface must be further evaluated. Thus, ongoing systematic investigations, including ex vivo, in vivo, and in vitro studies, are needed to evaluate the functionality and potential of this system. Moreover, controlled clinical studies will provide knowledge about the sufficiency of this combination system for the regeneration process and its effects on patient morbidity.

## 5. Conclusions

The present study analyzed the combination of an autologous liquid-PRF matrix as a drug delivery system, with five different xenogeneic collagen-based biomaterials histologically. Emphasis was placed on the distribution of leucocytes and platelets within the collagen membrane, and the interaction between the collagen membrane and liquid-PRF. Although all of the evaluated membranes were of porcine origin, three different types of interactions were observed, including total cellular penetration, partial cellular penetration, and no cellular penetration. The present study showed that it is possible to load biomaterials with a complex cell system (liquid-PRF) using a clinically applicable method within 15 min. PRF could serve as a drug delivery system to support GTR/GBR and enhance biomaterial bioactivity. Additionally, this ex vivo system could be used to assess the initial reactions of novel biomaterials, and thus reduce the number of animals used for in vivo studies. However, further investigations are required to evaluate the regeneration potential of this combination system and its clinical impact on patient morbidity.
